# Efficacy and toxicities of modified FOLFIRINOX versus gemcitabine plus nab-paclitaxel as first-line chemotherapy for unresectable pancreatic cancer: a systematic review and meta-analysis

**DOI:** 10.3389/fonc.2026.1913824

**Published:** 2026-09-28

**Authors:** Chunle Hu, Li Zhan, Yanhui Li, Xiawei Ren, Zhiyong Yu

**Affiliations:** 1 The First Clinical Institute, Zunyi Medical University, Zunyi, China; 2 School of Clinical Medicine, Southwest Medical University, Luzhou, China; 3 The First Clinical Medical College, Xinjiang Medical University, Urumqi, China; 4 Department of General Surgery, Affiliated Hospital of Zunyi Medical University, Zunyi, China; 5 Provincial Key Laboratory of Digestive System Diseases in Guizhou Province, Affiliated Hospital of Zunyi Medical University, Zunyi, China

**Keywords:** first-line chemotherapy, gemcitabine plus nab-paclitaxel, meta-analysis, modified FOLFIRINOX, overall survival, pancreatic cancer, unresectable

## Abstract

**Objective:**

Modified FOLFIRINOX (mFFX) and gemcitabine plus nab-paclitaxel (GnP) are both recommended as standard first-line regimens for unresectable pancreatic cancer (PC). However, direct head-to-head comparative evidence remains scarce, and prior studies have largely failed to distinguish mFFX from standard FOLFIRINOX (sFFX). We systematically compared mFFX with GnP to evaluate their efficacy and toxicities.

**Methods:**

We searched for randomized controlled trials (RCTs) and observational studies comparing mFFX with GnP as first-line therapy for unresectable PC published between January 2013 and May 2026. Overall survival (OS) and progression-free survival (PFS) were assessed using hazard ratios (HRs). Objective response rate (ORR) and disease control rate (DCR) were evaluated using risk ratios (RRs). Events of toxicities were assessed using odds ratios (ORs).

**Results:**

Five studies (three RCTs and two retrospective studies) involving 1204 patients (578 in the mFFX arm and 626 in the GnP arm) were included. Compared with GnP, mFFX was associated with a shorter OS (HR 1.26, 95% CI 1.09–1.45; *P* = 0.002) and PFS (HR 1.21, 95% CI 1.04–1.40; *P* = 0.01), along with a borderline lower DCR (RR 0.90, 95% CI 0.81–1.00; *P* = 0.05; I^2^ = 62%), which was consistent across RCTs (RR 0.88, 95% CI 0.81–0.94; I^2^ = 0%) but not observed in retrospective studies (RR 0.96, 95% CI 0.71–1.29; I^2^ = 87%), and ORR did not differ significantly between the two regimens. As for toxicity profiles, mFFX showed an elevated incidence of grade ≥3 diarrhea (OR 5.63) and anorexia (OR 4.44). By contrast, grade ≥3 neutropenia (OR 0.66) and white blood cell decrease (OR 0.49) occurred more frequently under GnP.

**Conclusions:**

GnP was associated with more favorable OS and PFS than mFFX, a borderline DCR advantage, and distinct toxicity profiles. Given the modest effect size and the limitations of this study, clinical decision-making should integrate patient age, performance status, and anticipated second-line therapy.

**Systematic review registration:**

https://www.crd.york.ac.uk/PROSPERO/, identifier CRD420251175959.

## 1 Introduction

Pancreatic cancer (PC) is among the most lethal malignancies worldwide, with a median overall survival of approximately 4 months (1) and a 5-year overall survival of merely 13% (2). In addition to this poor prognosis, its incidence has increased from 6.3 to 6.6 per 100,000 between 2010 and 2019 (3). It arises from glandular epithelium through precursor lesions to ultimately invasive carcinoma (2). Because of the insidious onset and rapid progression, the majority of patients (>80%) already present with locally advanced or metastatic disease by the time of diagnosis (4), and commonly experience recurrence following treatment. In this population, systemic chemotherapy continues to serve as the primary therapeutic modality (5), as immunotherapy and targeted therapy have shown limited efficacy.

For advanced PC, gemcitabine long served as the backbone of first-line chemotherapy. The PRODIGE 4/ACCORD 11 trial (6) demonstrated that FOLFIRINOX (the combination of oxaliplatin, irinotecan, leucovorin, fluorouracil) significantly prolonged median overall survival (OS) compared with gemcitabine monotherapy (11.1 vs. 6.8 months), although with increased toxicity. To improve tolerability, a modified FOLFIRINOX regimen (mFFX) was developed, in which the irinotecan dose was reduced and the 5-FU bolus omitted, preserving comparable survival benefits while substantially mitigating toxicities (7–10). Concurrently, the MPACT trial (11) established gemcitabine plus nab-paclitaxel (GnP) as another standard-of-care regimen, also demonstrating a survival benefit over gemcitabine monotherapy (median OS: 8.5 vs. 6.7 months). Regarding the comparison between FFX and GnP, prior studies have mostly evaluated standard-dose FFX (sFFX) or have not differentiated between dose intensity, rather than focusing on the mFFX regimen more widely adopted in current clinical practice; pooled evidence largely suggests that the survival benefit of sFFX is either superior or comparable to that of GnP (12, 13).

To date, evidence from direct head-to-head comparisons of mFFX versus GnP remains scarce. The recently published GENERATE (14) and PASS-01 (15) randomized controlled trials (RCTs) provided the first direct comparative evidence, yet their findings—that GnP numerically outperformed mFFX—stand in marked contrast to the widespread impression generated by numerous prior studies (13, 16) comparing sFFX with GnP. This discrepancy underscores the importance of distinguishing sFFX from mFFX and indicates that evidence derived solely from indirect comparisons or confounded by dose intensity can no longer adequately address the current clinical question. Therefore, this analysis aimed to comprehensively synthesize direct comparative data from RCTs and high-quality real-world studies, thereby providing the most up-to-date and definitive quantitative assessment of the efficacy and toxicity of mFFX versus GnP as contemporary first-line standards.

## 2 Materials and methods

### 2.1 Literature search

This meta-analysis was conducted in accordance with the Preferred Reporting Items for Systematic Reviews and Meta-Analyses (PRISMA) guidelines and was registered with PROSPERO (CRD420251175959). We systematically searched PubMed, Web of Science, Cochrane Central Register of Controlled Trials (CENTRAL), and Embase databases. The search strategy combined a pancreatic cancer block (‘pancreas cancer’/exp OR pancrea*:ti, ab, kw) with either of the two regimen blocks: mFOLFIRINOX (‘FOLFIRINOX’:ti, ab, kw OR (‘fluorouracil’/exp AND ‘leucovorin’/exp AND ‘irinotecan’/exp AND ‘oxaliplatin’/exp)) OR GnP (‘gemcitabine’/exp AND ‘albumin bound paclitaxel’/exp). Free-text terms including ‘unresectable’, ‘advanced’, and ‘first-line’ were also incorporated. The full search strategies for all databases are provided in the Supplementary Material. Two investigators (Chunle Hu, Li Zhan) independently screened titles, abstracts, and full texts; disagreements were resolved by consensus or through consultation with a third investigator.

### 2.2 Eligibility criteria

We included English-language RCTs or retrospective studies with an explicit comparative efficacy objective, published between January 2013 and May 2026, because this starting point marks the publication of the first standardized mFFX protocol by Mahaseth et al. (8). Studies were required to enroll patients aged ≥18 years with unresectable (locally advanced, metastatic, or recurrent) PC. For retrospective studies, dosing regimens had to be clearly specified: the intervention arm as mFFX (omitting the 5-FU bolus and with reduced irinotecan dose), and the control arm as standard GnP (administered on days 1, 8, and 15). Studies were required to report hazard ratios (HRs) and 95% confidence intervals (CIs) for OS or progression-free survival (PFS).

We excluded reviews, pathology reports, consensus papers, and studies not designed for efficacy comparison; trials using non-standard GnP; studies including patients with resectable disease or studies designed primarily for neoadjuvant or adjuvant therapy; non-first-line settings; trials where first-line treatment was combined with radiotherapy or other antineoplastic agents with intergroup imbalance and without adjustment; and retrospective studies without controlling for confounders through matching, weighting, or multivariable analysis.

### 2.3 Data extraction and quality assessment

We screened records against predefined criteria, with a third reviewer consulted to resolve disagreements. Records were initially assessed by title and abstract, followed by full-text review to confirm eligibility. Extracted items included first author, publication year, country, study period, sample size, patient characteristics, treatment details, median follow-up, efficacy outcomes, and safety data. The primary endpoint was OS. Secondary endpoints comprised PFS, objective response rate (ORR), disease control rate (DCR), and grade ≥3 toxicities. ORR was the proportion of complete response (CR) or partial response (PR) per Response Evaluation Criteria in Solid Tumors version 1.1. DCR was the proportion achieving CR, PR, or stable disease (SD). Grade ≥3 toxicities—including neutropenia, thrombocytopenia, anemia, anorexia, and diarrhea—were extracted per Common Terminology Criteria for Adverse Events version 4.0 or 5.0. ORR and DCR were analyzed in the response-evaluable population and toxicities in the safety population of each study; denominators therefore differ from the randomized sample sizes presented in **Table 1**.

**Table 1:** Treatment regimens of included studies.

Author year	Sample size (mFFX/GnP)	Details of mFFX	Details of GnP
Knox 2025 (15)	80/80	Per institutional standard of care	Per institutional standard of care
Ohba 2025 (14)	175/176	Oxaliplatin 85 mg/m^2^ + irinotecan 150mg/m^2^ + leucovorin 200 mg/m^2^ + fluorouracil 2400 mg/m^2^; every 2 weeks	Nab-paclitaxel 125 mg/m^2^ + gemcitabine 1000 mg/m^2^ on days 1, 8, 15; every 4 weeks
Ozaka 2023 (18)	62/63	Oxaliplatin 85 mg/m^2^ + irinotecan 150 mg/m^2^ + leucovorin 200 mg/m^2^ + fluorouracil 2400 mg/m^2^; every 2 weeks	Nab-paclitaxel 125 mg/m^2^ + gemcitabine 1000 mg/m^2^ on days 1, 8, 15; every 4 weeks
Ramaswamy 2023 (19)	152/198^†^	Oxaliplatin 65 mg/m^2^ + irinotecan 135mg/m^2^ + leucovorin 300 mg/m^2^ + fluorouracil 1800 mg/m^2^; every 2 weeks; mandatory G-CSF support	Nab-paclitaxel 125 mg/m^2^ + gemcitabine 1000 mg/m^2^ on days 1, 8, 15; every 4 weeks
Mie 2021 (20)	109/109	Oxaliplatin 85 mg/m^2^ + irinotecan 150 mg/m^2^ + leucovorin 200 mg/m^2^ + fluorouracil 2400 mg/m^2^; every 2 weeks; irinotecan reduced to 67% for *UGT1A1* HM/CH	Nab-paclitaxel 125 mg/m^2^ + gemcitabine 1000 mg/m^2^ on days 1, 8, 15; every 4 weeks

^†^For Ramaswamy 2023, the full cohort comprised 350 patients (152 mFFX/198 GnP); the OS hazard ratio was extracted from the multivariable Cox proportional hazards model of this cohort (the only adjusted survival estimate reported); ORR, DCR, and grade ≥3 toxicities were extracted from the 1:1 propensity-matched cohort (130 per arm). Both adjustment and matching accounted for age >60 years and ECOG PS 2.

mFFX, modified FOLFIRINOX (the combination of oxaliplatin, irinotecan, leucovorin, fluorouracil, with the fluorouracil bolus omitted and at least irinotecan dose reduced); GnP, gemcitabine plus nab-paclitaxel; *UGT1A1*, UDP-glucuronosyltransferase 1A1; HM, homozygous; CH, compound heterozygous (*6 and *28 variants); G-CSF, granulocyte colony-stimulating factor.

Risk of bias was evaluated using the Cochrane Risk of Bias tool version 2.0 (RoB 2.0) (17) for RCTs and the Newcastle-Ottawa Scale (NOS) for observational studies. NOS scores of ≥7, 5–6, and ≤4 were considered high, moderate, and low quality, respectively. Two reviewers independently performed the assessments; discrepancies were resolved through discussion.

### 2.4 Statistical analysis

All meta-analyses in this study primarily employed a fixed-effects model, a choice prespecified on the rationale that, after strict application of inclusion and exclusion criteria, the included studies demonstrated high consistency in intervention dosing, treatment schedule, and comparator definition. In the presence of substantial heterogeneity (I² ≥ 50%), the prespecified subgroup analysis by study design (RCT vs. retrospective) was conducted to explore its source, and a random-effects model was used for pooling. Sensitivity analyses by sequential exclusion of individual studies were performed to assess the robustness of the pooled results.

OS and PFS were analyzed using HRs with 95% CIs; risk ratios (RRs) were used for ORR and DCR; and odds ratios (ORs) were used for grade ≥3 toxicities. HRs were pooled using the inverse-variance method, and RRs and ORs using the Mantel-Haenszel method. Heterogeneity was quantified with the I² statistic. An I² value ≥ 50% was considered indicative of substantial heterogeneity. Publication bias was assessed by Egger’s test when ≥10 studies were included. Pooled analyses were performed using RevMan 5.3, and leave-one-out sensitivity analyses were conducted using Stata 12.0. Two-sided *P* < 0.05 was considered statistically significant.

## 3 Results

### 3.1 Study selection and characteristics

The search retrieved 7864 records; after deduplication, 3541 were screened by title and abstract. 12 full-text articles were assessed for eligibility, and 5 studies met the inclusion criteria (**Figure 1**). These comprised three RCTs—PASS-01 (Knox 2025) (15) from North America, GENERATE (Ohba 2025) (14) and JCOG1407 (Ozaka 2023) (18) from Japan—and two retrospective studies from India (Ramaswamy 2023) (19) and Japan (Mie 2021) (20). A total of 1204 patients were included (578 mFFX; 626 GnP); median age ranged 55–67 years, and 59.6% were male. Metastatic disease was present in 75.5% and liver metastases in 56.6%. Baseline characteristics and study designs are summarized in **Table 2**, and treatment regimens are presented in **Table 1**. The GnP regimen was uniform across all five studies (nab-paclitaxel 125 mg/m² plus gemcitabine 1, 000 mg/m² on days 1, 8, and 15, every 4 weeks), whereas the mFFX regimen varied. Three studies (14, 18, 20) used identical starting doses (oxaliplatin 85 mg/m², irinotecan 150 mg/m², leucovorin 200 mg/m², and fluorouracil 2, 400 mg/m² as a 46-hour infusion without bolus, every 2 weeks); in Mie et al. (20), irinotecan was further reduced to 67% of the starting dose in patients homozygous or compound heterozygous for UGT1A1*6/*28. Ramaswamy et al. (19) used lower starting doses (oxaliplatin 65 mg/m², irinotecan 135 mg/m², leucovorin 300 mg/m², fluorouracil 1, 800 mg/m²) with mandatory primary G-CSF prophylaxis. In PASS-01 (15), mFFX was dosed per institutional standards and the specific doses were not reported. All 3 RCTs were rated as having some concerns with RoB 2.0; the 2 retrospective studies scored 8–9 on the NOS, indicating high quality (**Supplementary Tables S1**, **S2**).

**Figure 1:**
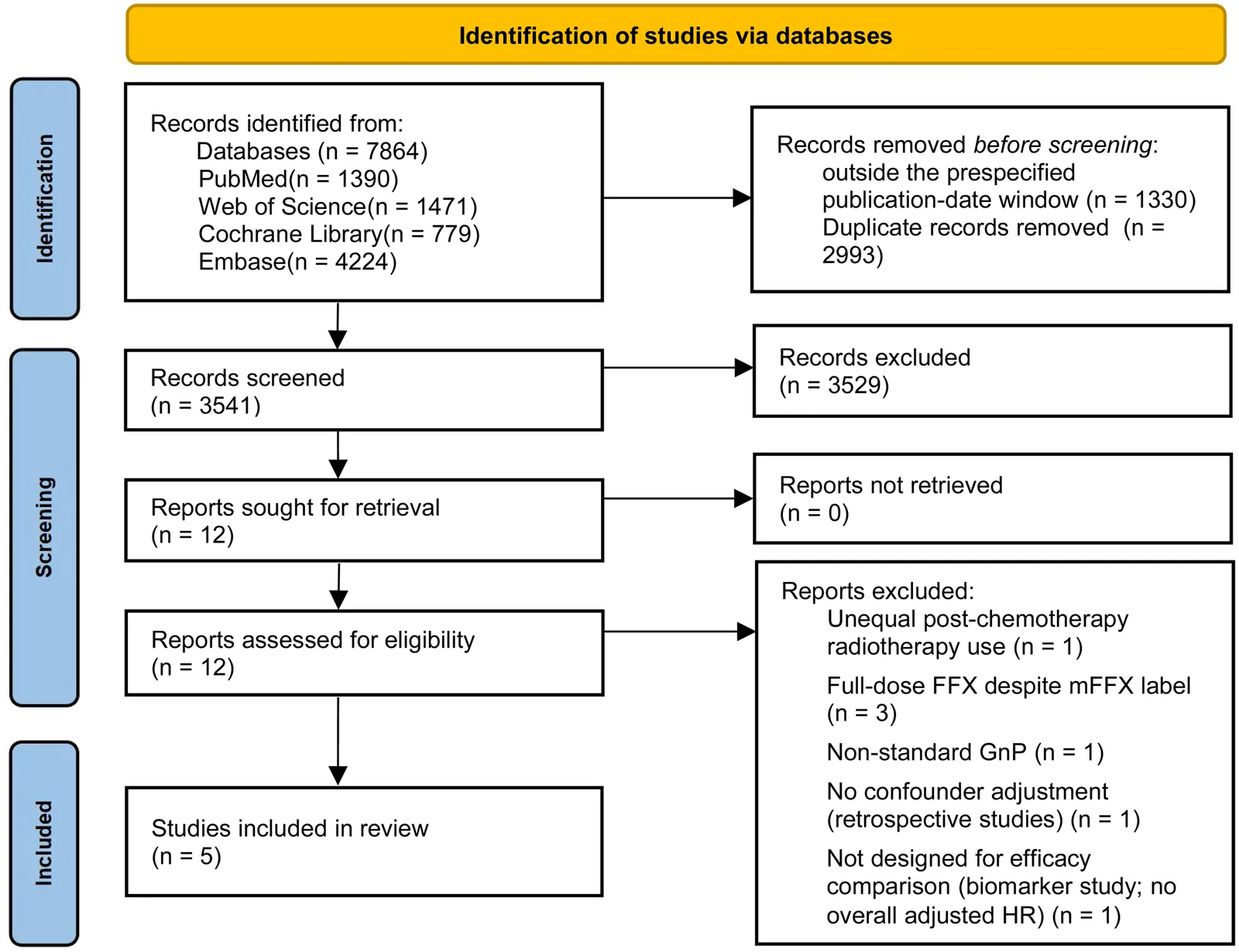
Study selection flowchart (PRISMA diagram).

**Table 2:** Baseline characteristics and study designs of included studies.

Author year	Country	Study period	Study design	Disease status	Response evaluation criteria	Safety criteria	Median Age	ECOG score	Liver metastasis	ClinicalTrials.gov/ethics
Knox 2025 (15)	US, Canada	2020.10-2024.1	RCT (Phase II)	M	RECIST 1.1	CTCAE 5.0	64	0/1	83.8%	NCT04469556
Ohba 2025 (14)	Japan	2019.4-2023.3	RCT (Phase II/III)	M/Rec	RECIST 1.1	CTCAE 4.0	67/65	0/1	68.7%	jRCTs031190009
Ozaka 2023 (18)	Japan	2016.7-2019.8	RCT (Phase II)	LA	RECIST 1.1	CTCAE 4.0	66	0/1	NA	UMIN000023143
Ramaswamy 2023 (19)	India	2013.1-2019.12	Retrospective	M/LA	RECIST 1.1	CTCAE 4.0	55	0-2	48.3%	IEC 900655
Mie 2021 (20)	Japan	2014.3-2019.12	Retrospective	M/Rec	RECIST 1.1	CTCAE 4.0	61/62	0-2	63.3%	2020-1123

RCT, randomized controlled trial; RECIST, Response Evaluation Criteria in Solid Tumors; CTCAE, Common Terminology Criteria for Adverse Events; ECOG, Eastern Cooperative Oncology Group; M, metastatic; LA, locally advanced; Rec, recurrent; NA, not applicable.

In retrospective studies specifically, for Ramaswamy et al. (19), the HR for OS was extracted from a multivariable Cox proportional hazards model of the full cohort (n = 350), adjusted for age >60 years and ECOG performance status 2 (the only adjusted survival estimate reported); dichotomous outcomes such as ORR, DCR, and grade ≥3 toxicities, for which no adjusted estimates were reported, were derived from the 1:1 propensity-matched cohort (130 patients per arm) matched on the same two variables. For Mie et al. (20), no multivariable model was reported; instead, HRs for OS and PFS and dichotomous outcomes were all derived from the 1:1 propensity score-matched cohort (109 patients per arm), with age, sex, performance status, serum carcinoembryonic antigen, serum CA 19-9, and disease status (metastatic or recurrent) balanced between groups through matching. Thus, all HRs from these two studies were estimates controlled for baseline confounders, derived through multivariable adjustment or propensity score matching, and were used as the primary effect sizes for pooling.

### 3.2 OS

All 5 studies (14, 15, 18–20) reported OS data. Under a fixed-effects model, the pooled analysis demonstrated that mFFX was associated with a shorter OS compared with GnP in patients with unresectable PC, with a combined HR of 1.26 (95% CI: 1.09–1.45; *P* = 0.002) (**Figure 2**). Between-study heterogeneity was negligible (I^2^ = 0%, *P* = 0.68).

**Figure 2:**
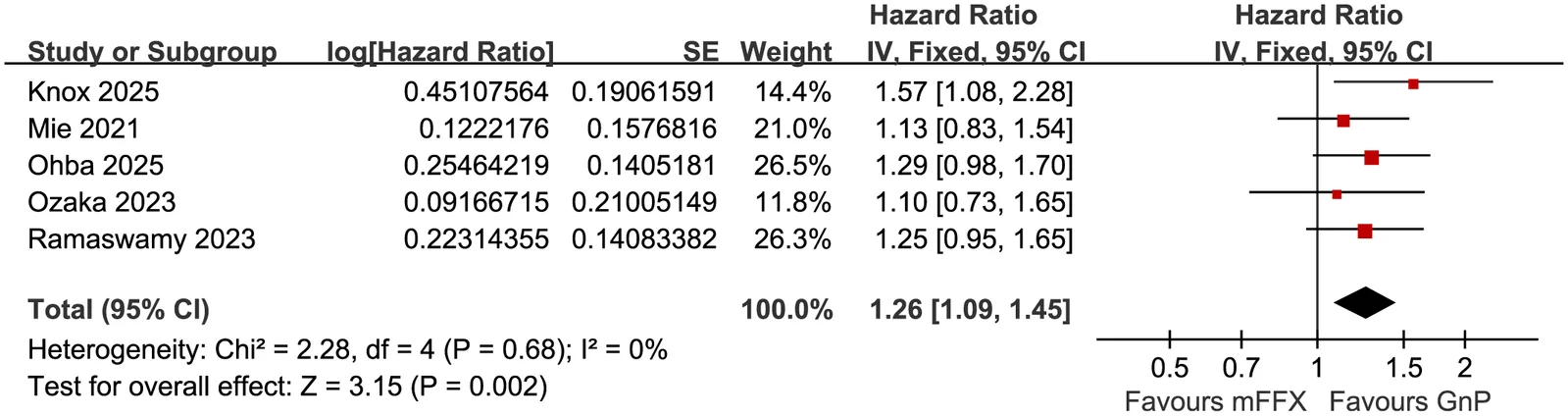
Forest plot of OS. CI, confidence interval; OS, overall survival; mFFX, modified FOLFIRINOX (the combination of oxaliplatin, irinotecan, leucovorin, fluorouracil, with the fluorouracil bolus omitted and at least irinotecan dose reduced); GnP, gemcitabine plus nab-paclitaxel.

Because OS is susceptible to post-progression therapy, the potential influence of the between-arm differences in second-line treatment was assessed. Second-line therapy was reported in all five studies (**Supplementary Table S3**). Between-arm differences in the proportion of patients receiving second-line chemotherapy were non-significant in every study. Leave-one-out sensitivity analysis showed no individual study with significant impact on the overall pooled effect estimate and confirmed robustness (**Supplementary Figure S1**), including omission of the study with the largest between-arm difference in second-line receipt (Ozaka 2023: HR 1.28, 95% CI 1.10–1.49) and of the only study with more second-line therapy proportion in the GnP arm (Knox 2025: HR 1.21, 95% CI 1.04–1.41).

### 3.3 PFS

Four studies (14, 15, 18, 20) reported PFS data; one retrospective study (19) did not provide a PFS HR and was excluded from the analysis. The combined estimate indicated that mFFX was associated with a shorter PFS compared with GnP in patients with unresectable PC, with a pooled HR of 1.21 (95% CI: 1.04–1.40; *P* = 0.01) (**Figure 3**). No statistical heterogeneity was observed (I^2^ = 0%, *P* = 0.69). Leave-one-out analysis showed that the pooled estimate lost statistical significance upon omission of Knox 2025 (HR 1.17, 95% CI 0.99–1.38; *P* = 0.06), and narrowly retained significance upon omission of Ozaka 2023 (HR 1.18, 95% CI 1.00–1.39; *P* = 0.04), although the direction of the point estimates remained consistent throughout (**Supplementary Figure S2**).

**Figure 3:**
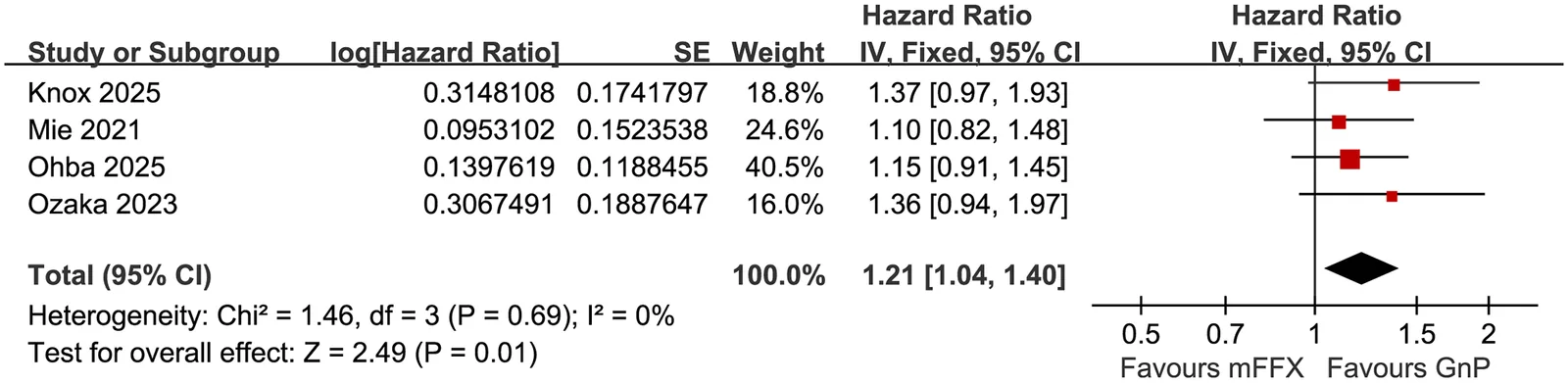
Forest plot of PFS. CI, confidence interval; PFS, progression-free survival; mFFX, modified FOLFIRINOX (the combination of oxaliplatin, irinotecan, leucovorin, fluorouracil, with the fluorouracil bolus omitted and at least irinotecan dose reduced); GnP, gemcitabine plus nab-paclitaxel.

### 3.4 ORR and DCR

Evaluable ORR data were available from all 5 studies (14, 15, 18–20). Pooled analysis under a fixed-effects model detected no significant ORR disparity between GnP and mFFX in first-line chemotherapy of unresectable PC (RR 0.86, 95% CI: 0.73–1.01; *P* = 0.07), though the point estimate indicated a lower ORR with mFFX relative to GnP. Heterogeneity was absent (I^2^ = 0%, *P* = 0.65). Ramaswamy et al. (19) was the only study showing a statistically significant ORR advantage for GnP (RR 0.73, 95% CI: 0.54–0.99) (**Figure 4A**).

**Figure 4:**
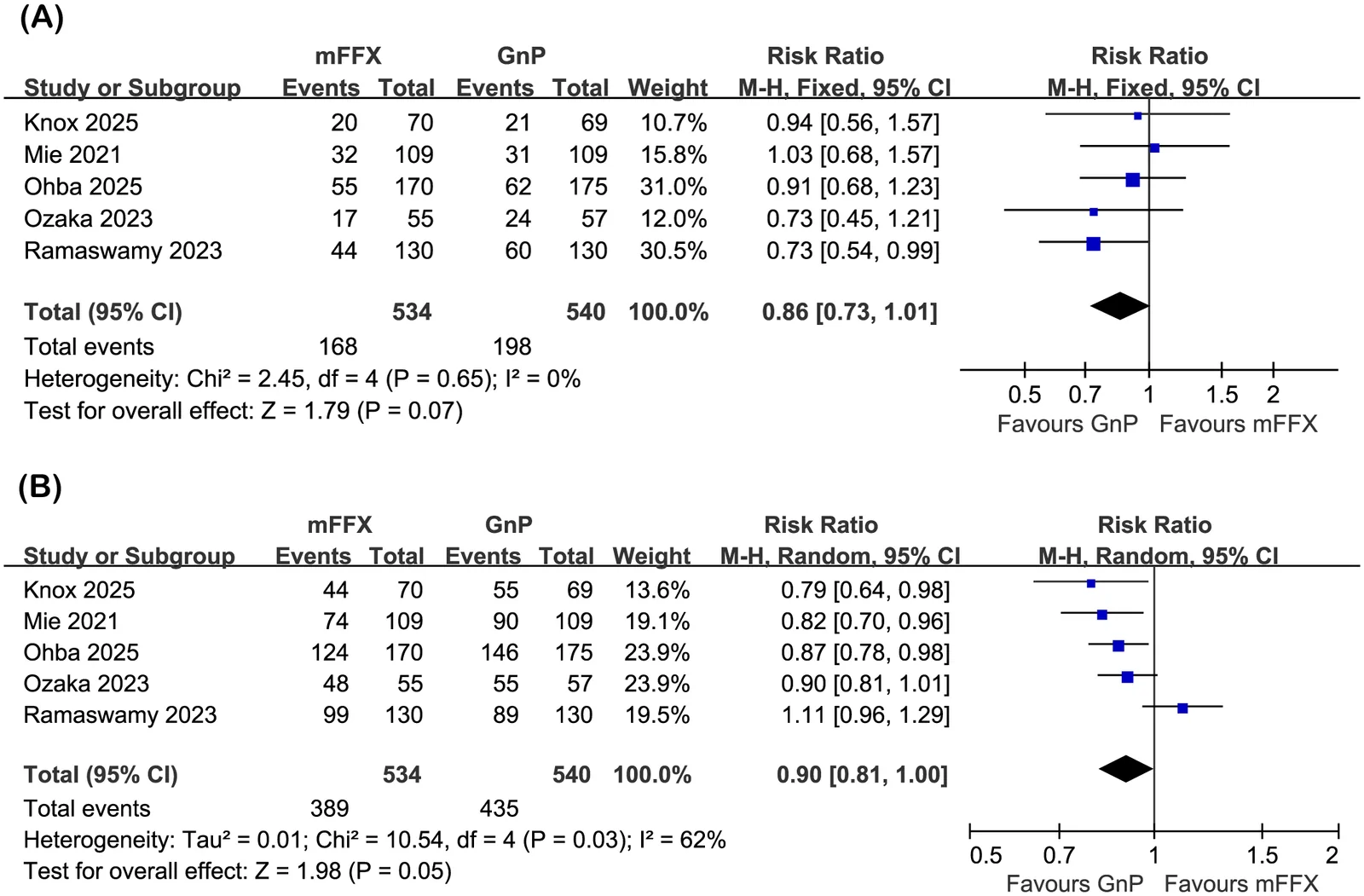
Forest plot of ORR **(A)** and DCR **(B)**. ORR and DCR were analyzed in the response-evaluable population of each study, denominators therefore differ from the randomized sample sizes presented in **Table 1**. CI, confidence interval; ORR, objective response rate; DCR, disease control rate; mFFX, modified FOLFIRINOX (the combination of oxaliplatin, irinotecan, leucovorin, fluorouracil, with the fluorouracil bolus omitted and at least irinotecan dose reduced); GnP, gemcitabine plus nab-paclitaxel.

For DCR, all five studies were pooled under a random-effects model, as prespecified for outcomes with substantial heterogeneity. The pooled analysis showed a borderline lower DCR with mFFX compared with GnP (RR 0.90, 95% CI 0.81–1.00; *P* = 0.05), with substantial between-study heterogeneity (I^2^ = 62%, *P* = 0.03) (**Figure 4B**). Subgroup analysis by study design was performed to explore the source of heterogeneity (**Supplementary Figure S3**). In the RCT subgroup (14, 15, 18), mFFX was consistently associated with a significantly lower DCR (RR 0.88, 95% CI 0.81–0.94; *P* = 0.0006), without heterogeneity (I^2^ = 0%). In contrast, the retrospective subgroup (19, 20) showed no significant difference between regimens (RR 0.96, 95% CI 0.71–1.29; *P* = 0.77), with substantial heterogeneity (I^2^ = 87%) driven by the divergent effect direction of Ramaswamy et al. (RR 1.11, 95% CI 0.96–1.29) (19). The test for subgroup differences was not statistically significant (*P* = 0.57); given the limited number of studies within each subgroup, this analysis should be interpreted as exploratory. These findings indicate that the heterogeneity in DCR originated primarily from the retrospective evidence, whereas the RCT data consistently favored GnP.

### 3.5 Toxicity

Grade ≥3 adverse event data were provided by 4 studies (14, 18–20); Knox et al. (15) reported only serious adverse events and was excluded from toxicity pooling. The two regimens exhibited markedly distinct toxicity profiles.

In terms of hematologic toxicities, among the 4 studies reporting grade ≥3 neutropenia, the incidence was significantly lower with mFFX than with GnP (OR 0.66, 95% CI: 0.50–0.87; *P* = 0.003; I^2^ = 0%) (**Figure 5A**). Two studies (14, 18) reported data on grade ≥3 white blood cell decrease, and the pooled analysis similarly favored mFFX (OR 0.49, 95% CI: 0.32–0.73; I^2^ = 0%) (**Figure 5B**). No statistically significant differences were found for grade ≥3 anemia, thrombocytopenia, or febrile neutropenia (**Supplementary Figure S4**).

**Figure 5:**
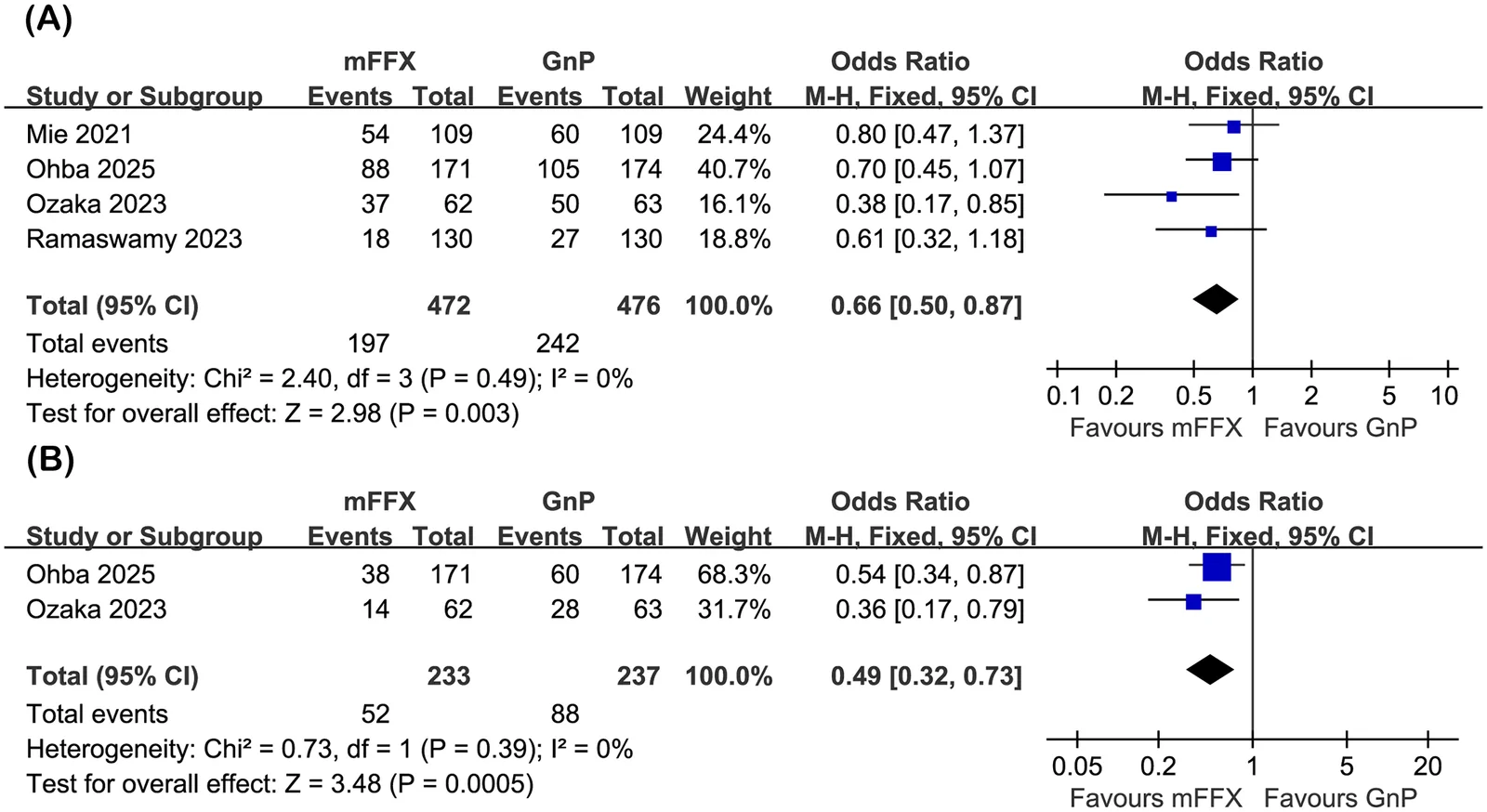
Forest plot of Grade ≥3 hematological toxicities: Neutropenia **(A)**, White Blood Cell Decrease **(B)**. Toxicities were analyzed in the safety population of each study, denominators therefore differ from the randomized sample sizes presented in **Table 1**. CI, confidence interval; mFFX, modified FOLFIRINOX (the combination of oxaliplatin, irinotecan, leucovorin, fluorouracil, with the fluorouracil bolus omitted and at least irinotecan dose reduced); GnP, gemcitabine plus nab-paclitaxel.

In terms of non-hematologic toxicities, across the 4 studies reporting grade ≥3 diarrhea, risk was markedly elevated with mFFX (OR 5.63, 95% CI: 3.05–10.40; *P* < 0.00001; I^2^ = 0%) (**Figure 6A**). Three studies (14, 18, 20) reported data on grade ≥3 anorexia, and the pooled analysis also showed higher grade ≥3 anorexia with mFFX (OR 4.44, 95% CI: 2.58–7.65; I^2^ = 0%) (**Figure 6B**). No significant differences were found for grade ≥3 infection, ALT/AST increase, fatigue, nausea, or neuropathy (**Supplementary Figure S5**).

**Figure 6:**
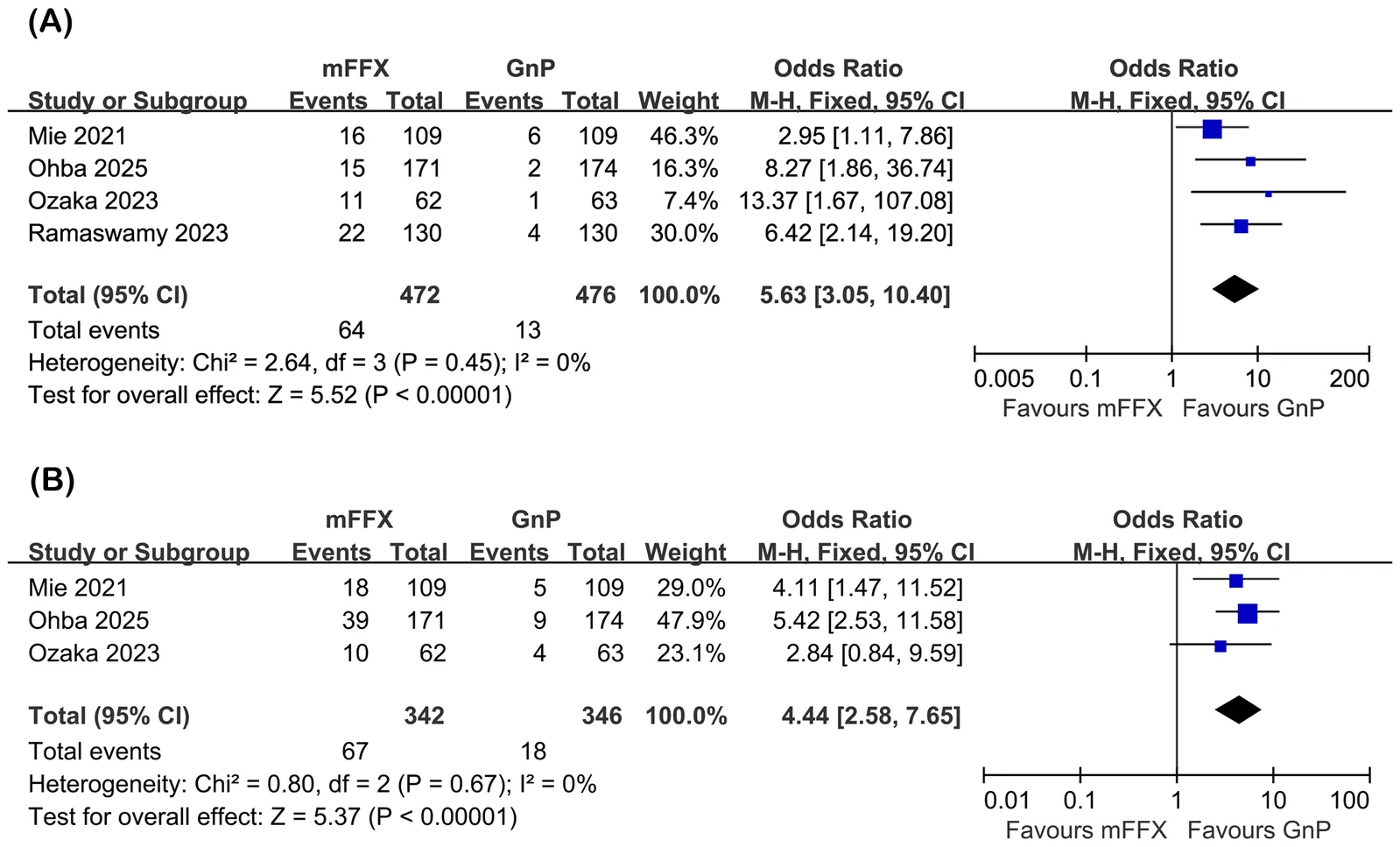
Forest plot of Grade ≥3 non-hematological toxicities: Diarrhea **(A)**, Anorexia **(B)**. Toxicities were analyzed in the safety population of each study, denominators therefore differ from the randomized sample sizes presented in **Table 1**. CI, confidence interval; mFFX, modified FOLFIRINOX (the combination of oxaliplatin, irinotecan, leucovorin, fluorouracil, with the fluorouracil bolus omitted and at least irinotecan dose reduced); GnP, gemcitabine plus nab-paclitaxel.

### 3.6 Risk of bias and heterogeneity assessment

Three RCTs (14, 15, 18) were rated as “some concerns” in the RoB 2.0 assessment, primarily due to the open-label design. Two retrospective studies (19, 20) achieved NOS scores of 8–9, indicating high quality. No significant heterogeneity was observed for the OS (I^2^ = 0%, *P* = 0.68), PFS (I^2^ = 0%, *P* = 0.69), or ORR, supporting the robustness of fixed-effects model estimates. DCR showed substantial heterogeneity (I^2^ = 62%) across five studies and was therefore pooled using a random-effects model; subgroup analysis attributed this to the retrospective subgroup, driven by Ramaswamy et al. (19)—the only study whose point estimate favored mFFX—which received only one star in the NOS comparability domain owing to residual imbalance after propensity matching (**Supplementary Table S2**). Publication bias assessment was not performed given fewer than 10 studies. Sensitivity analysis demonstrated stable pooled effect sizes for OS, which is the primary endpoint.

## 4 Discussion

Head-to-head RCTs directly comparing mFFX with GnP are extremely scarce (21), yet both regimens are recommended as standard options by ESMO (22) and NCCN (23) guidelines, leaving a long-standing gap in direct evidence to guide regimen selection. Prior comparative studies often failed to distinguish mFFX from sFFX when comparing “FOLFIRINOX” with GnP (24, 25), despite differences in toxicity profiles, efficacy, and cost that may arise from mFFX’s elimination of the 5-FU bolus and reduced irinotecan dosing (26). This study strictly defined the dose and cycle of mFFX, excluding any full-dose regimens, thereby ensuring homogeneity of the intervention.

Given the paucity of RCTs, high-quality retrospective studies were incorporated to supplement the evidence. In most such studies, the mFFX cohort tended to be younger with better Eastern Cooperative Oncology Group performance status (ECOG PS) (27, 28), generating spurious survival advantages in unadjusted analyses (29). Adjusted analyses in Ramaswamy et al. (19) and Mie et al. (20) both substantiated this: after controlling for confounders, mFFX no longer demonstrated survival superiority, and Ohba et al. (14)’s subgroup analysis also showed that the OS advantage of GnP was further enhanced among patients aged ≥65 years. This phenomenon essentially reflects selection bias arising from the clinical choice of regimen based on performance status, rather than evidence of superior antitumor activity of mFFX. ESMO guidelines explicitly recommend GnP for ECOG PS 2 patients (22), which supports this interpretation. Accordingly, to ensure analytic reliability, retrospective study eligibility was strictly restricted regarding research objectives, baseline comparability, and bias control methodology.

Pooled analysis under these inclusion criteria revealed that mFFX was associated with a shorter OS (HR 1.26, 95% CI 1.09–1.45) and PFS (HR 1.21, 95% CI 1.04–1.40) compared with GnP, with consistent direction across studies; mFFX also showed a borderline lower DCR (RR 0.90, 95% CI 0.81–1.00) with substantial heterogeneity (I^2^ = 62%), although the difference in ORR was not significant. The only study directionally favoring mFFX and the source of the heterogeneity for DCR was Ramaswamy et al. (19), whose divergence is plausibly explained by residual imbalance: younger, fitter, and non-metastatic patients had been preferentially assigned to mFFX, and matching restricted to age and performance status left locally advanced disease over-represented in the mFFX arm (44% vs 20% P<0.001), though metastatic stage was not significantly associated with OS in their univariate analysis (*P* = 0.301); such tumors may achieve disease control more readily than metastatic ones. Toxicity profiles differed markedly: mFFX carried higher risk of grade ≥3 diarrhea and anorexia, whereas GnP was distinguished by more grade ≥3 neutropenia and white blood cell decrease.

Interpretation of these pooled findings must consider factors potentially modifying effect estimates. At the enrollment level, mFFX efficacy may have been systematically underestimated in included studies. Knox et al. (15)’s study, conducted in Western populations, excluded germline *BRCA1/2* and *PALB2* mutation carriers—who account for 10–15% of PC (30) and are most sensitive to platinum-based therapy (31). The remaining four studies were conducted in Asian populations. Population-level data indicate that the prevalence of germline BRCA mutations in Asian populations is approximately half that in White populations (32), a difference hypothesized by Lee et al. (33) to partially explain the more favorable outcomes of FOLFIRINOX in Western studies and the comparable or even greater efficacy of GnP reported in Asian cohorts. However, germline BRCA status was largely unexamined in these studies: only Ohba et al. (14) performed testing in no more than half of their cohort, whereas the other three did not report BRCA mutation data at all. Whether and to what extent this genetic background influenced our pooled results therefore remains uncertain. Meanwhile, Ohba et al. (14), Ozaka et al. (18), and Mie et al. (20) all enrolled *UGT1A1* mutation carriers, who are intolerant of irinotecan (34, 35). The absence of platinum-responsive populations along with the inclusion of irinotecan-intolerant populations may have somewhat compromised mFFX’s overall performance in trials. Concurrently, Kaplan-Meier curves from Ozaka et al. (18), Mie et al. (20), and Ramaswamy et al. (19) displayed a consistent pattern: GnP curves positioned superiorly early, with crossover around 6 months after which mFFX assumed dominance, suggesting that early-intolerant patients on mFFX exited rapidly, while long-term survivors may derive more durable benefit.

Beyond these population-level factors, overall survival is also shaped by what happens after progression. In four of the five included studies, the proportion of patients receiving second-line therapy was nonsignificantly higher in the mFFX arm than in the GnP arm (all P > 0.2), with PASS-01 as the sole exception in direction (55% vs 61%, *P* = 0.48) (**Supplementary Table S3**) (15). Whether patients proceed to second-line therapy is not inconsequential for OS: in Ramaswamy et al. (19), patients who received second-line chemotherapy survived significantly longer than those who did not (median OS 14.46 vs 9.17 months; P < 0.001), and in an analysis (36) of the randomized MPACT population, receipt of second-line therapy was independently associated with both longer total OS (HR 0.50; P < 0.001) and longer post-first-line survival (HR 0.47; P < 0.001). However, receiving second-line therapy requires surviving to progression in a condition fit enough to tolerate it, so recipients are a positively selected subgroup whose longer survival may reflect not only a benefit of treatment but also the effect of this selection. Against this background, we conducted the sensitivity analysis of the pooled OS estimate, and it remained stable throughout (**Supplementary Figure S1**).

Beyond the proportion of recipients, the two regimens also differed in what patients could receive after progression. First-line mFFX simultaneously commits three active drug classes (a fluoropyrimidine, irinotecan, and oxaliplatin), leaving gemcitabine-based regimens as the principal second-line option—for which intensification (with paclitaxel) of the gemcitabine backbone has not further prolonged survival in an RCT after FOLFIRINOX failure (37)—whereas first-line GnP commits only gemcitabine and a taxane, preserving more drug classes for later use, for which intensification (with nanoliposomal irinotecan or oxaliplatin) of the fluorouracil backbone has further prolonged survival in phase III trials after gemcitabine-based therapy (38, 39). The discrepancy of regimen selection is inherent to any head-to-head comparison of the two regimens and cannot be neutralized by study design, such that the second-line regimens reported in the included studies all followed this pattern (**Supplementary Table S3**). In Ohba et al. (14), median PFS was more comparable between arms (5.8 vs 6.7 months) than OS (14.0 vs 17.0 months). The investigators explicitly attributed this separation to the post-progression period, reporting that post-progression survival was longer in the GnP group (5.5 vs 7.0 vs 5.6 months for mFFX, GnP, and S-IROX; log-rank *P* = 0.03) and was “likely the cause of the OS difference”. However, in Knox et al. (15), post-progression survival did not differ significantly between arms (4.2 vs 4.7 months; *P* = 0.12), although the point estimate favored GnP. This between-trial difference may be partly explained by the use of liposomal irinotecan, the agent whose addition to a fluorouracil backbone prolonged survival in the phase III trial (38) mentioned above. In Ohba et al. (14), it accounted for 34.3% of second-line regimens in the GnP arm but only 1.8% in the mFFX arm, plausibly because first-line mFFX had already delivered irinotecan; in Knox et al. (15), however, second-line regimens were not reported in detail.

Taken together, the OS difference observed in the overall population should be regarded as a composite outcome shaped by multiple effect modifiers, rather than as a property of the first-line agents alone. As second-line therapy in the included studies differed between arms in both receipt and composition, the pooled OS difference is better understood as a feature of two sequential treatment strategies and should not be taken as evidence of an intrinsic, class-level superiority of one first-line regimen in any patient subgroup over the other.

Of note, the present OS findings favoring GnP diverge from prior meta-analyses focused on sFFX. The pooled analysis by Merza et al. (12) of 21 observational studies suggested comparable OS and potentially superior PFS with sFFX versus GnP (HR: 0.88, 95% CI: 0.81-0.97; *P* < 0.0001), while Nichetti et al. (13)’s network meta-analysis of RCTs indicated a survival advantage of sFFX over GnP for both PFS and OS. This discrepancy may be attributed to several factors. First, key RCTs had not been published at the time of their search cutoff. Second, baseline imbalances in retrospective studies tended to overestimate FFX efficacy, whereas in our meta-analysis, the eligibility criteria used in the original studies (excluding germline *BRCA1/2* and *PALB2* carriers and including *UGT1A1* mutation carriers) may have underestimated mFFX performance. Chen et al. (40) conducted an indirect treatment comparison between mFFX and GnP in their network meta-analysis, the results of which were consistent in direction with our findings. Their reported point estimates favored GnP for OS and PFS, with statistically significant ORR superiority of GnP over mFFX (RR: 1.43; 95% CI: 1.04–1.96). Lee et al. (41)’s ethnic-stratified meta-analysis further revealed that Western populations derived superior survival benefit from FOLFIRINOX (HR 0.84, 95% CI 0.74–0.95), whereas the Asian subgroup demonstrated significantly better overall survival with GnP (HR 1.29, 95% CI 1.03–1.60). The primary studies included in our present analysis were predominantly Asian cohorts, with pooled results directionally consistent with Lee et al. (41)’s Asian subgroup conclusion. It must be acknowledged, however, that despite the statistically significant OS advantage for GnP in this meta-analysis, the effect size is modest (pooled HR 1.26). Whether such a difference can change clinical practice in a malignancy with an extremely poor prognosis, such as PC, remains open to discussion.

Beyond survival outcomes, the two regimens exhibit distinct toxicity profiles. This meta-analysis demonstrated significantly higher grade ≥3 diarrhea incidence with mFFX, consistent with established pharmacologic mechanisms and prior knowledge. Direct damage to the intestinal mucosal epithelium by SN-38, the active metabolite of irinotecan (42), is its dose-limiting toxicity. In mFFX, despite reduced irinotecan dosing, diarrhea remains a distinguishing adverse event with a markedly higher incidence than GnP (OR 5.63), reflecting the persistent role of SN-38-mediated mucosal injury. For grade ≥3 neutropenia, our analysis showed significantly higher rates with GnP—a finding in contrast to PRODIGE 4/ACCORD 11 (6) and Pusceddu et al. (43)’s studies including sFFX. Plausible explanations include: mFFX’s reduced irinotecan dosing (especially a further 67% dose reduction for *UGT1A1* mutation carriers (20)), elimination of the 5-FU bolus, and mandatory prophylactic G-CSF support in one study (19), collectively reducing myelosuppressive intensity.

Conventional wisdom derived from sFFX data tended to associate it with substantial hematological toxicity (6, 12, 13, 43). However, this analysis suggests that with standardized supportive care and pharmacogenomic screening, the relative hematological toxicity profile of mFFX versus GnP may diverge from sFFX-based assumptions. Taken together, clinical decision-making should not rely solely on population-averaged survival benefits, but must integrate patient age, ECOG PS, individual toxicity tolerance, hepatic metastasis burden, anticipated second-line course, and cost-effectiveness considerations for each regimen.

Given the variation in starting doses detailed above, planned dose intensities in the three identically dosed studies (14, 18, 20) were 75, 42.5, and 1, 200 mg/m2/week for irinotecan, oxaliplatin, and fluorouracil, compared with values 10–25% lower in Ramaswamy et al. (19) (67.5, 32.5, and 900 mg/m^2^/week), also the only study specifying mandatory primary G-CSF prophylaxis. Delivered dose intensities in the mFFX arms, reported in the two Japanese RCTs (14, 18), were similar between them (irinotecan 74.9% and 75.3%; oxaliplatin 78.9% and 76.9%; fluorouracil 86.4% and 85.5% of planned) and were not lower than in the corresponding GnP arms (nab-paclitaxel 68.5% and 72.2%; gemcitabine 75.4% and 79.8%); in both trials, the survival outcomes favoring GnP thus occurred despite comparable delivered dose intensity. Of note, the only study showing a significantly lower ORR with mFFX (RR 0.73) was also the one with the lowest starting doses (19), although its DCR estimate directionally favored mFFX (RR 1.11), and the contribution of dose intensity to these divergent findings remains unclear. For toxicities, grade ≥3 neutropenia in both arms was markedly less frequent in Ramaswamy et al. (19)—the only study using lower starting doses—than in the three studies using identical higher doses (14, 18, 20) (mFFX 13.8% vs 51.5%, 59.7%, 49.5%; GnP 20.8% vs 60.3%, 79.4%, 55.0%), which may reflect the lower dose intensity, the mandatory G-CSF prophylaxis, or even more plausibly population- and reporting-level differences, given that both arms of this only non-Japanese study were affected, whereas grade ≥3 diarrhea varied (8.8–17.7%) without an apparent relation to dose. For the remaining three studies, delivered dose intensity was not reported, leaving the relationship between dose intensity and outcomes open to speculation.

Several limitations warrant caution when interpreting our findings. First, the small number of included studies and the predominantly Asian study populations—coupled with limited Western representation—may limit the generalizability of our results. Second, mFFX dose-reduction strategies varied slightly across studies: three used identical starting doses and only one used lower doses (19), with one RCT administering mFFX per institutional standards without reporting specific doses (15), precluding complete equivalence assumptions, and delivered dose intensity was available from only two studies (14, 18); with so few studies and dose variation limited to a single study (19), a formal meta-regression of dose intensity against treatment effects was not feasible, and any contribution of dose variation to the pooled estimates remains unquantified. Third, post-progression therapy could not be accounted for in the pooled analysis: both the proportion of patients receiving second-line treatment and the regimens they received differed between arms, such that the pooled OS difference may partly reflect the sequential treatment strategies rather than the first-line regimens alone. Fourth, two nuances in the adjusted estimates warrant acknowledgment: the HRs from Mie et al. were univariable estimates from a propensity-matched cohort with well-balanced baseline characteristics, while for Ramaswamy et al., the adjusted HR was obtained from the multivariable analysis of the unmatched full cohort because the matched-pair analysis did not report an HR; moreover, adjustment was limited to age and ECOG PS, leaving possible residual confounding by unbalanced factors such as disease stage and hepatic metastasis, which may also have affected the dichotomous outcomes. Fifth, neither molecular subtype nor germline BRCA status was systematically assessed across the included studies, precluding any pooled or subgroup analysis by these factors; whether treatment effects differ by transcriptomic subtype, and whether ethnic differences in genetic background contributed to the pooled results, therefore remain unknown. Nevertheless, this study represents the first pooled analysis based on direct head-to-head comparisons suggesting more favorable OS, PFS with GnP over mFFX. Given the restricted accessibility and cost barriers facing novel regimens such as NALIRIFOX (44), clarifying the relative value of existing standards is of urgent importance. Future research should explore predictive biomarkers (45) and integrate transcriptomic subtyping (46) with DNA damage repair status (30) into composite molecular stratification (47) for prospective trials, enabling precision optimization of first-line chemotherapy based on individualized toxicity risk profiles.

## 5 Conclusions

This meta-analysis provides the first direct comparative evidence that GnP may demonstrate advantages over mFFX in OS and PFS, with a borderline DCR advantage that was consistent across randomized trials but not corroborated by retrospective studies, while ORR did not differ significantly. The two regimens have markedly distinct toxicity profiles: mFFX carries a higher risk of grade ≥3 diarrhea and anorexia, while GnP is associated with more grade ≥3 neutropenia and white blood cell decrease. Although GnP demonstrates an overall population advantage, the effect size is modest and may be modified by multiple factors. Clinical decision-making should therefore integrate patient age, performance status, and anticipated second-line therapy to enable individualized treatment selection.

## Data Availability

The original contributions presented in the study are included in the article/**Supplementary Material**. Further inquiries can be directed to the corresponding author.
