# Supplementary material for: Efficacy and toxicities of modified FOLFIRINOX versus gemcitabine plus nab-paclitaxel as first-line chemotherapy for unresectable pancreatic cancer: a systematic review and meta-analysis
Source: Front Oncol. 2026 Sep 28;16:1913824. doi: 10.3389/fonc.2026.1913824 (PMC13655002; doi:10.3389/fonc.2026.1913824)
Supplement: Supplementary file 1 [file SupplementaryFile1.docx]

Supplementary Material

**Detailed search strategy**

**PubMed**

#1 "Pancreatic Neoplasms"[Mesh] OR pancrea*[tiab]

#2 unresectable[tiab] OR inoperable[tiab] OR advanced[tiab] OR metastatic[tiab] OR "locally advanced"[tiab] OR "Stage IV"[tiab]

#3 "first-line"[tiab] OR "first line"[tiab] OR "initial therap*"[tiab] OR "treatment-naive"[tiab] OR "previously untreated"[tiab] OR "palliative chemotherap*"[tiab] OR "palliative intent"[tiab]

#4 "FOLFIRINOX"[tiab] OR "FOLFOXIRI"[tiab] OR "mFOLFIRINOX"[tiab] OR "mFOLFOXIRI"[tiab] OR (("Fluorouracil"[MeSH] OR "Fluorouracil"[tiab] OR "5-FU"[tiab]) AND ("Leucovorin"[MeSH] OR "Leucovorin"[tiab] OR "Folinic Acid"[tiab]) AND ("Irinotecan"[MeSH] OR "Irinotecan"[tiab]) AND ("Oxaliplatin"[MeSH] OR "Oxaliplatin"[tiab]))

#5 "Gemcitabine"[MeSH] OR "Gemcitabine"[tiab] OR "Albumin-Bound Paclitaxel"[MeSH] OR "nab-paclitaxel"[tiab] OR "nab paclitaxel"[tiab] OR "GnP"[tiab] OR ("Gemcitabine"[tiab] AND ("nab-paclitaxel"[tiab] OR "Albumin-Bound Paclitaxel"[tiab]))

#6 #1 AND #2 AND #3 AND (#4 OR #5)

**Cochrane**

#1 [mh "Pancreatic Neoplasms"] OR pancrea*:ti,ab,kw

#2 unresectable:ti,ab,kw OR inoperable:ti,ab,kw OR advanced:ti,ab,kw OR metastatic:ti,ab,kw OR "locally advanced":ti,ab,kw OR "Stage IV":ti,ab,kw

#3 "first-line":ti,ab,kw OR "first line":ti,ab,kw OR "initial therapy":ti,ab,kw OR "initial therapies":ti,ab,kw OR "treatment-naive":ti,ab,kw OR "previously untreated":ti,ab,kw OR "palliative chemotherapy":ti,ab,kw OR "palliative intent":ti,ab,kw

#4 FOLFIRINOX:ti,ab,kw OR FOLFOXIRI:ti,ab,kw OR mFOLFIRINOX:ti,ab,kw OR mFOLFOXIRI:ti,ab,kw OR (([mh "Fluorouracil"] OR Fluorouracil:ti,ab,kw OR "5-FU":ti,ab,kw OR "5 FU":ti,ab,kw) AND ([mh "Leucovorin"] OR Leucovorin:ti,ab,kw OR "Folinic Acid":ti,ab,kw) AND ([mh "Irinotecan"] OR Irinotecan:ti,ab,kw) AND ([mh "Oxaliplatin"] OR Oxaliplatin:ti,ab,kw))

#5 [mh "Gemcitabine"] OR Gemcitabine:ti,ab,kw OR [mh "Albumin-Bound Paclitaxel"] OR "nab-paclitaxel":ti,ab,kw OR "nab paclitaxel":ti,ab,kw OR GnP:ti,ab,kw OR (Gemcitabine:ti,ab,kw AND ("nab-paclitaxel":ti,ab,kw OR "Albumin-Bound Paclitaxel":ti,ab,kw))

#6 #1 AND #2 AND #3 AND (#4 OR #5)

**EMBASE**

#1 'pancreas cancer'/exp OR 'pancreas carcinoma'/exp OR 'pancreas adenocarcinoma'/exp OR 'pancreas tumor'/exp OR pancrea*:ti,ab,kw

#2 unresectable:ti,ab,kw OR inoperable:ti,ab,kw OR advanced:ti,ab,kw OR metastatic:ti,ab,kw OR 'locally advanced':ti,ab,kw OR 'Stage IV':ti,ab,kw

#3 'first-line':ti,ab,kw OR 'first line':ti,ab,kw OR 'initial therapy':ti,ab,kw OR 'initial therapies':ti,ab,kw OR 'treatment-naive':ti,ab,kw OR 'previously untreated':ti,ab,kw OR 'palliative chemotherapy':ti,ab,kw OR 'palliative intent':ti,ab,kw

#4 'folfirinox':ti,ab,kw OR 'folfoxiri':ti,ab,kw OR 'mfolfirinox':ti,ab,kw OR 'mfolfoxiri':ti,ab,kw OR (('fluorouracil'/exp OR fluorouracil:ti,ab,kw OR '5-FU':ti,ab,kw OR '5 FU':ti,ab,kw) AND ('leucovorin'/exp OR leucovorin:ti,ab,kw OR 'folinic acid':ti,ab,kw) AND ('irinotecan'/exp OR irinotecan:ti,ab,kw) AND ('oxaliplatin'/exp OR oxaliplatin:ti,ab,kw))

#5 'gemcitabine'/exp OR gemcitabine:ti,ab,kw OR 'albumin bound paclitaxel'/exp OR 'nab-paclitaxel':ti,ab,kw OR 'nab paclitaxel':ti,ab,kw OR 'GnP':ti,ab,kw OR (gemcitabine:ti,ab,kw AND ('nab-paclitaxel':ti,ab,kw OR 'albumin bound paclitaxel':ti,ab,kw))

#6 #1 AND #2 AND #3 AND (#4 OR #5)

**WEB OF SCIENCE**

#1 TS=(pancrea*)

#2 TS=(unresectable OR inoperable OR advanced OR metastatic OR "locally advanced" OR "Stage IV")

#3 TS=("first-line" OR "first line" OR "initial therapy" OR "initial therapies" OR "treatment-naive" OR "previously untreated" OR "palliative chemotherapy" OR "palliative intent")

#4 TS=(FOLFIRINOX OR FOLFOXIRI OR mFOLFIRINOX OR mFOLFOXIRI) OR (TS=(Fluorouracil OR "5-FU" OR "5 FU") AND TS=(Leucovorin OR "Folinic Acid") AND TS=(Irinotecan) AND TS=(Oxaliplatin))

#5 TS=(Gemcitabine OR "nab-paclitaxel" OR "nab paclitaxel" OR GnP) OR TS=("Albumin-Bound Paclitaxel") OR (TS=(Gemcitabine) AND TS=("nab-paclitaxel" OR "nab paclitaxel" OR "Albumin-Bound Paclitaxel"))

#6 #1 AND #2 AND #3 AND (#4 OR #5)

**Table S1.** Quality assessment of included RCTs using the Cochrane Risk of Bias 2.0 (RoB 2.0) tool.

| Study | Randomisation process | Deviations from intended interventions | Missing outcome data | Measurement of the outcome | Selection of the reported result | Overall Risk of Bias |
| --- | --- | --- | --- | --- | --- | --- |
| Ozaka 2023 | Low | Low | Low | Some concerns | Low | Some concerns |
| Knox 2025 | Some concerns | Some concerns | Low | Some concerns | Low | Some concerns |
| Ohba 2025 | Low | Low | Low | Some concerns | Low | Some concerns |

**Table S2.** Quality assessment of included retrospective cohort studies using the Newcastle-Ottawa Scale (NOS).

| Cohort Studies | Selection | | | | Comparability | Outcome | | | Score |
| --- | --- | --- | --- | --- | --- | --- | --- | --- | --- |
|  | Representativeness of the exposed cohort | Selection of the  non-exposed cohort | Ascertainment  of exposure | Demonstration that outcome  of interest was not present  at start of study | Comparability of cohorts on the basis of the design or analysis | Assessment  of outcome | Was follow-up long enoughfor outcomes to occur? | Adequacy of  follow-up of cohorts |  |
| Ramaswamy 2023 | ★ | ★ | ★ | ★ | ★ | ★ | ★ | ★ | 8 |
| Mie 2021 | ★ | ★ | ★ | ★ | ★★ | ★ | ★ | ★ | 9 |

**Table S3.** Second-line therapy after disease progression in the five included studies.

| first author year | Category | Measure | GnP arm | mFFX arm | Δ (mFFX−GnP, pp) | P value |
| --- | --- | --- | --- | --- | --- | --- |
| Ohba 2025 | second-line therapy | Yes, n/N (%) | 105/176 (59.7%) | 111/175 (63.4%) | +3.8 | 0.54 |
|  |  | No, n (%) | 71 (40.3%) | 64 (36.6%) | — | — |
|  | regimen | Fluoropyrimidine-based | 50 (47.6%) | 32 (28.8%) |  |  |
|  |  | Gemcitabine-based | 17 (16.2%) | 75 (67.6%) |  |  |
|  |  | Liposomal irinotecan–based | 36 (34.3%) | 2 (1.8%) |  |  |
|  |  | Chemoradiotherapy | 2 (1.9%) | 1 (0.9%) |  |  |
|  |  | Other | 0 (0%) | 1 (0.9%) |  |  |
| Knox 2025 | second-line therapy | Yes, % | 61% | 55% | −6.0 | 0.48 |
|  |  | No, % | 39% | 45% | — | — |
| Ramaswamy 2023 | second-line therapy | Yes, n/N (%) | 89/198 (44.9%) | 78/152 (51.3%) | +6.4 | 0.28 |
|  |  | No, n (%) | 109 (55.1%) | 74 (48.7%) | — | — |
|  | regimen | Most common regimens | CAPIRI / FOLFIRI | GnP / gemcitabine-based |  |  |
| Ozaka 2023 | second-line therapy | Yes, n/N (%) | 47/63 (74.6%) | 51/62 (82.3%) | +7.7 | 0.41 |
|  |  | No, n (%) | 16 (25.4%) | 11 (17.7%) | — | — |
|  | regimen | mFFX | 14/47 (29.8%) | NA |  |  |
|  |  | GnP | NA | 21/51 (41.2%) |  |  |
| Mie 2021 | second-line therapy | Yes, n/N (%) | 76/109 (69.7%) | 78/109 (71.6%) | +1.8 | 0.88 |
|  |  | No, n (%) | 33 (30.3%) | 31 (28.4%) | — | — |
|  | regimen | mFFX | 39 (51.3%) | 0 (0%) |  |  |
|  |  | GnP | 0 (0%) | 55 (70.5%) |  |  |
|  |  | mFOLFIRI | 2 (2.6%) | 0 (0%) |  |  |
|  |  | Gemcitabine monotherapy | 0 (0%) | 9 (11.5%) |  |  |
|  |  | IRIS (irinotecan + S-1) | 0 (0%) | 1 (1.3%) |  |  |
|  |  | S-IROX | 1 (1.3%) | 1 (1.3%) |  |  |
|  |  | S-1 | 26 (34.2%) | 5 (6.4%) |  |  |
|  |  | Conversion surgery | 6 (7.9%) | 5 (6.4%) |  |  |
|  |  | Clinical trial | 2 (2.6%) | 2 (2.6%) |  |  |

Δ: between-arm difference in the proportion receiving second-line therapy (mFFX minus GnP), in percentage points; NA: not available; GnP: gemcitabine plus nab-paclitaxel; mFFX: modified FOLFIRINOX; mFOLFIRI: modified fluorouracil, leucovorin, and irinotecan; CAPIRI: capecitabine plus irinotecan; FOLFIRI: fluorouracil, leucovorin, and irinotecan; IRIS: irinotecan plus S-1; S-IROX: S-1, irinotecan, and oxaliplatin; S-1: tegafur–gimeracil–oteracil.

**Figure S1.** Leave-one-out sensitivity analysis of the pooled OS estimate. Each row presents the pooled hazard ratio recalculated after omitting one study at a time, using the same fixed-effects model as the primary analysis. OS: overall survival.

**Figure S2.** Leave-one-out sensitivity analysis of the pooled PFS estimate. Each row presents the pooled hazard ratio recalculated after omitting one study at a time, using the same fixed-effects model as the primary analysis. PFS: progression-free survival.

**Figure S3.** Subgroup analysis of DCR by study design. DCR were analyzed in the response-evaluable population of each study, denominators therefore differ from the randomized sample sizes presented in Table 1. RCT, randomized controlled trial; CI: confidence interval; DCR: disease control rate; mFFX: modified FOLFIRINOX (the combination of oxaliplatin, irinotecan, leucovorin, fluorouracil, with the fluorouracil bolus omitted and at least irinotecan dose reduced); GnP: gemcitabine plus nab-paclitaxel.

**Figure S4.** Forest plot of Grade ≥3 hematological toxicities without statistically significant difference between mFFX and GnP: Anemia (A), Thrombocytopenia (B), Febrile Neutropenia (C). Toxicities were analyzed in the safety population of each study, denominators therefore differ from the randomized sample sizes presented in Table 1. CI: confidence interval; mFFX: modified FOLFIRINOX (the combination of oxaliplatin, irinotecan, leucovorin, fluorouracil, with the fluorouracil bolus omitted and at least irinotecan dose reduced); GnP: gemcitabine plus nab-paclitaxel.

**Figure S5.** Forest plot of Grade ≥3 non-hematological toxicities without statistically significant difference between mFFX and GnP: Infection (A), ALT/AST Increase (B), Fatigue (C), Nausea (D), Neuropathy (E). Toxicities were analyzed in the safety population of each study, denominators therefore differ from the randomized sample sizes presented in Table 1. CI: confidence interval; mFFX: modified FOLFIRINOX (the combination of oxaliplatin, irinotecan, leucovorin, fluorouracil, with the fluorouracil bolus omitted and at least irinotecan dose reduced); GnP: gemcitabine plus nab-paclitaxel. Random-effects models were used for outcomes with substantial heterogeneity (I^2^ ≥ 50%: A, D); fixed-effects models were used otherwise.
